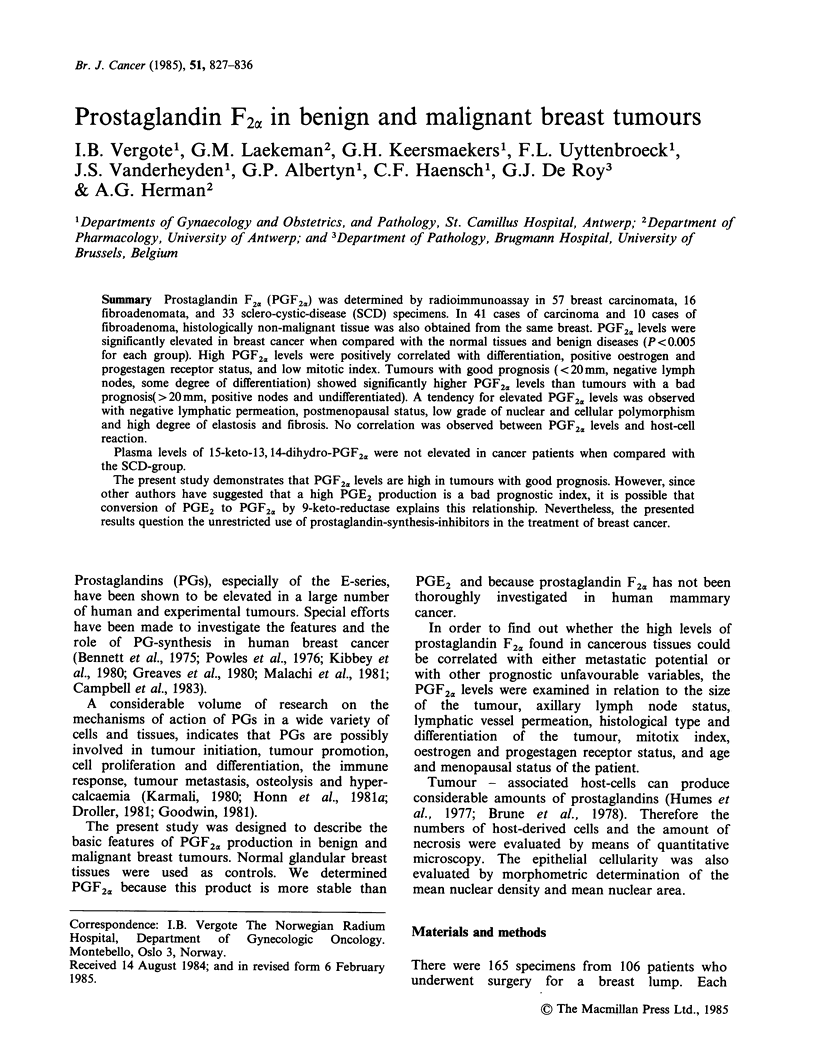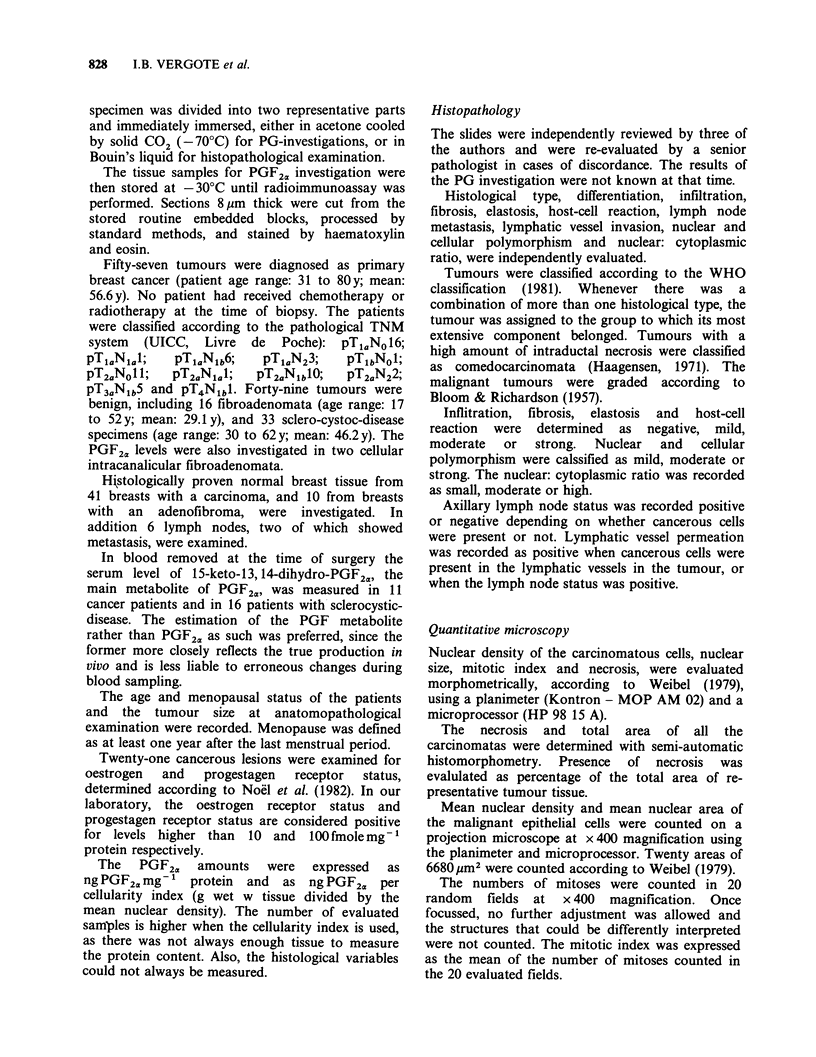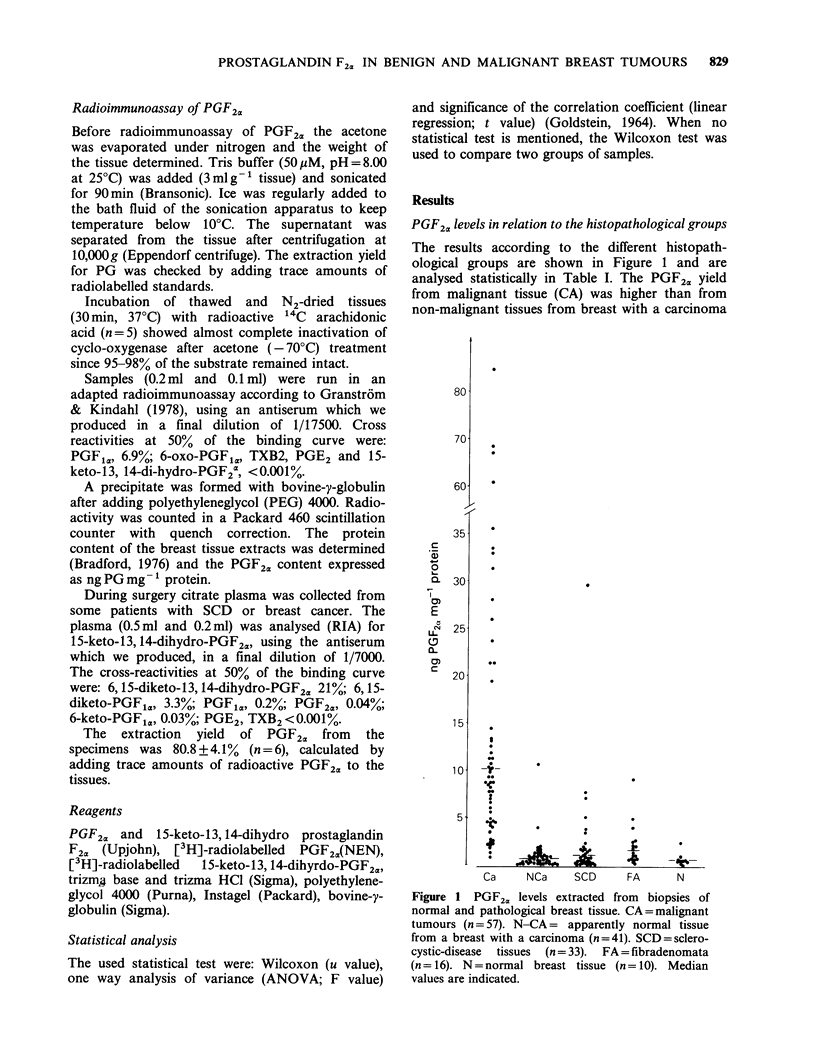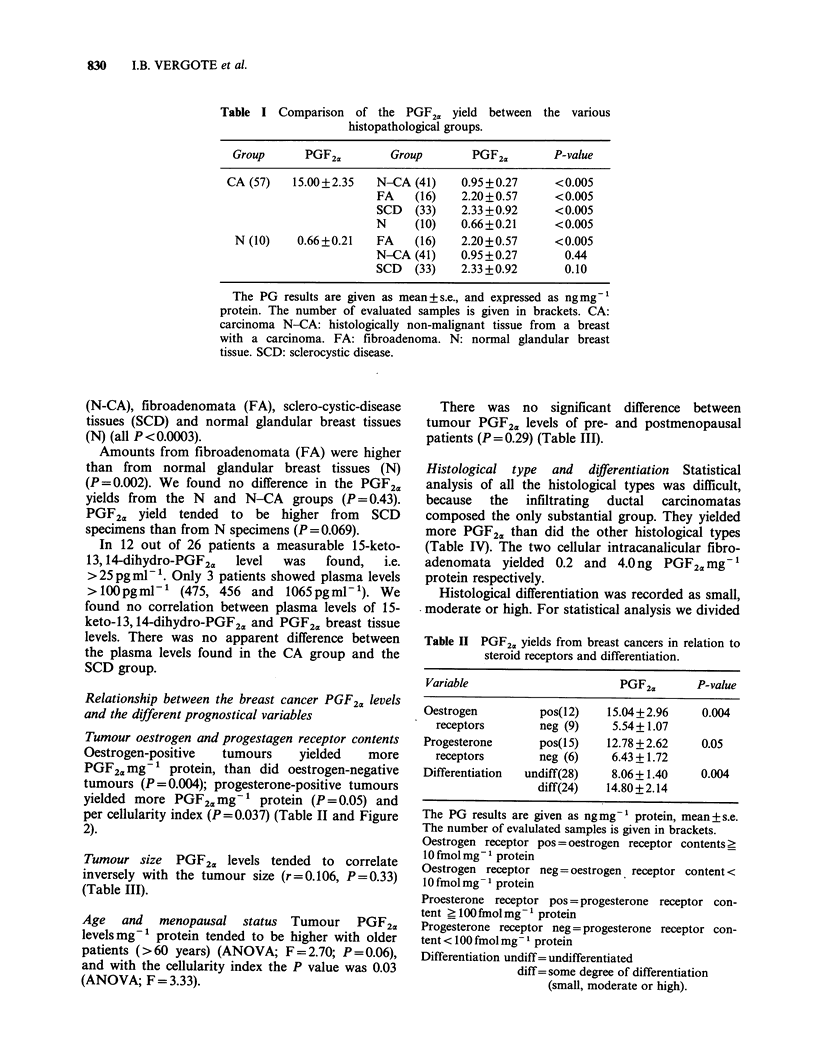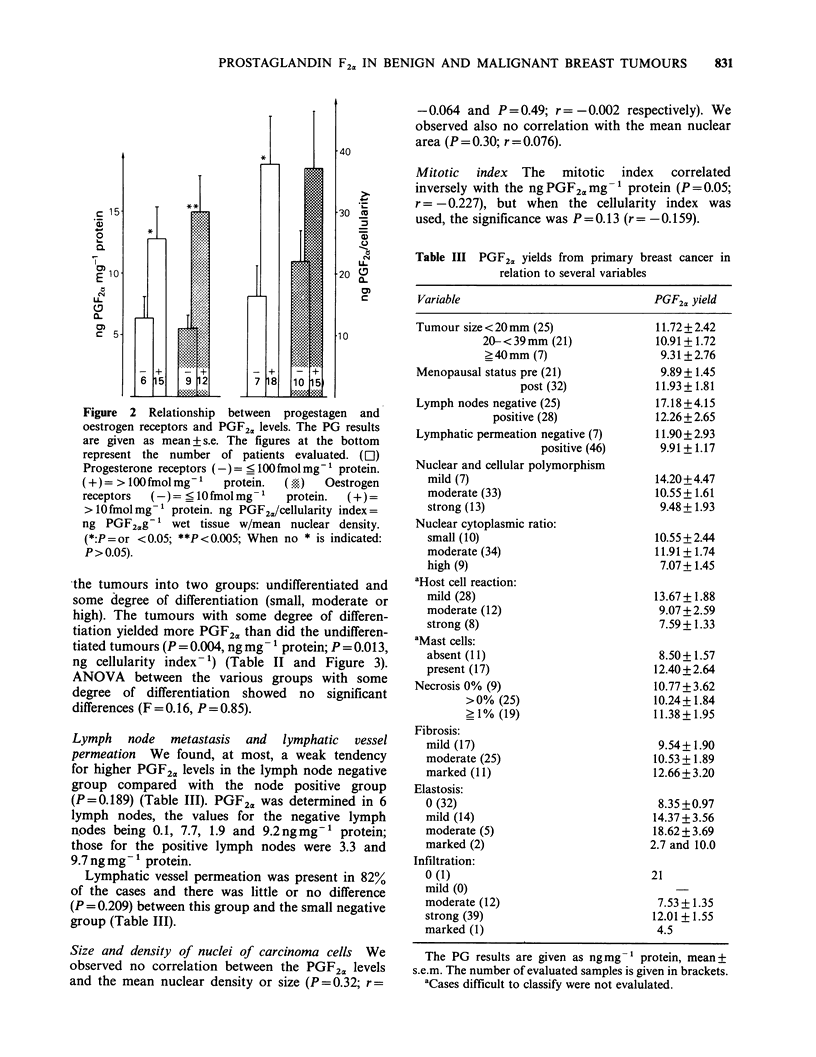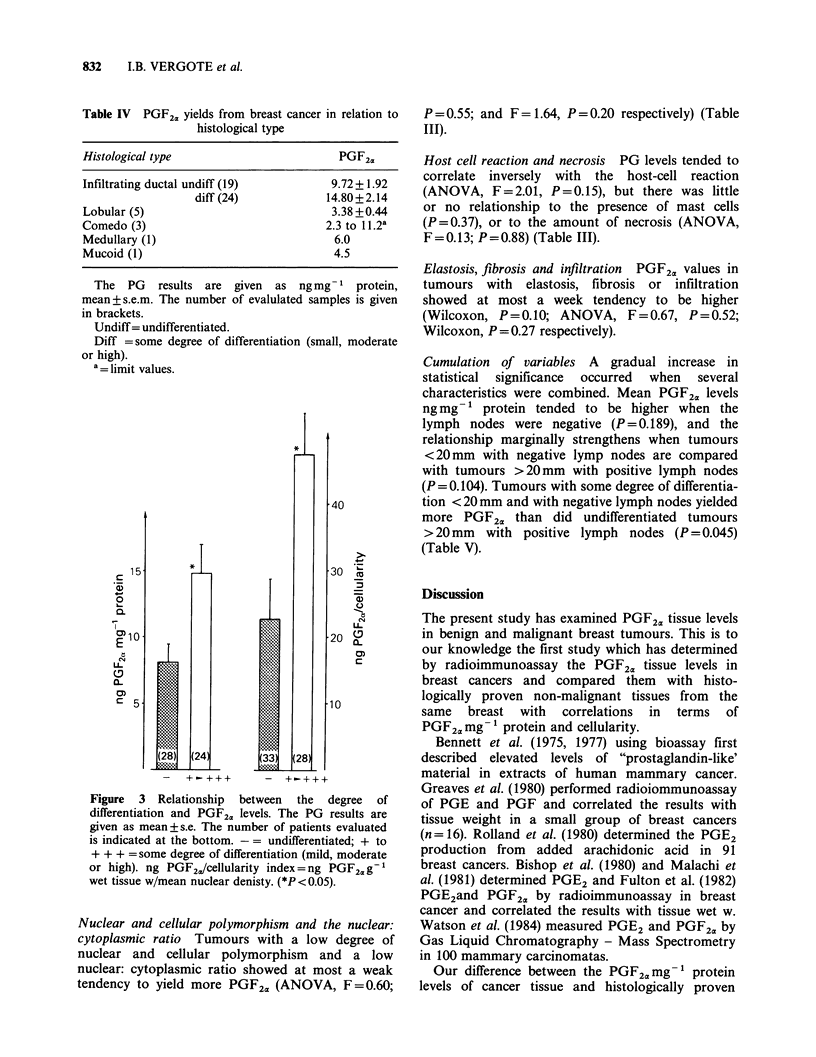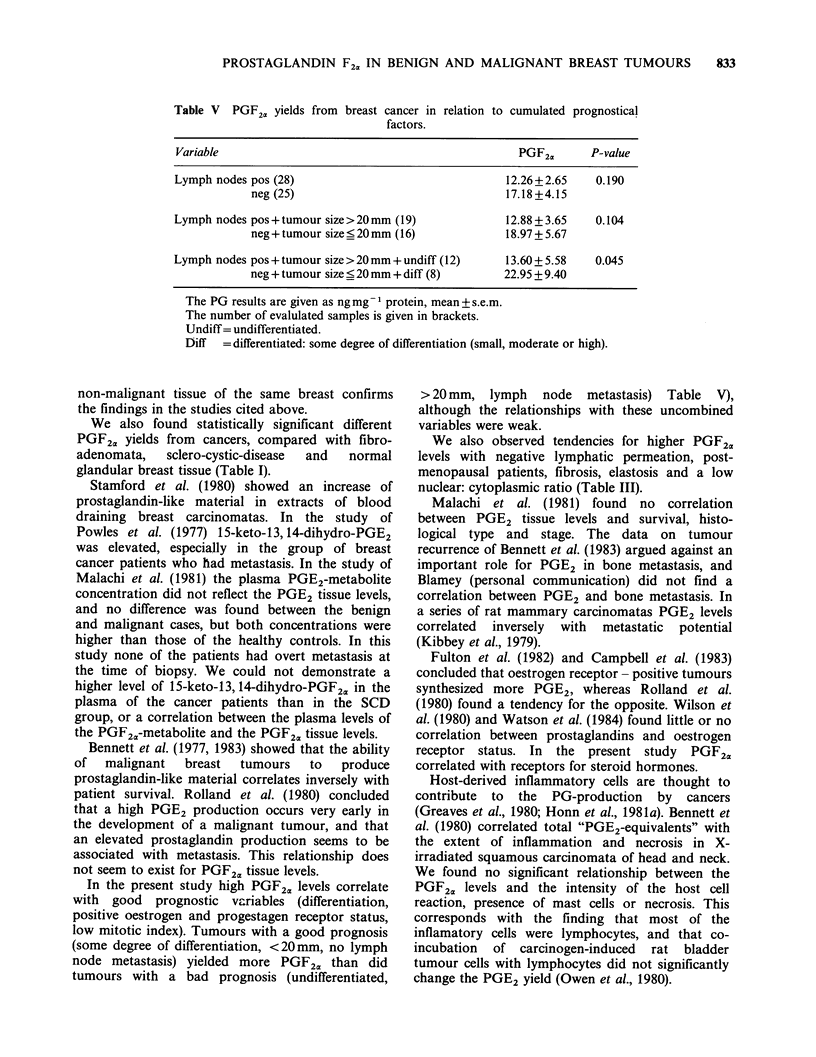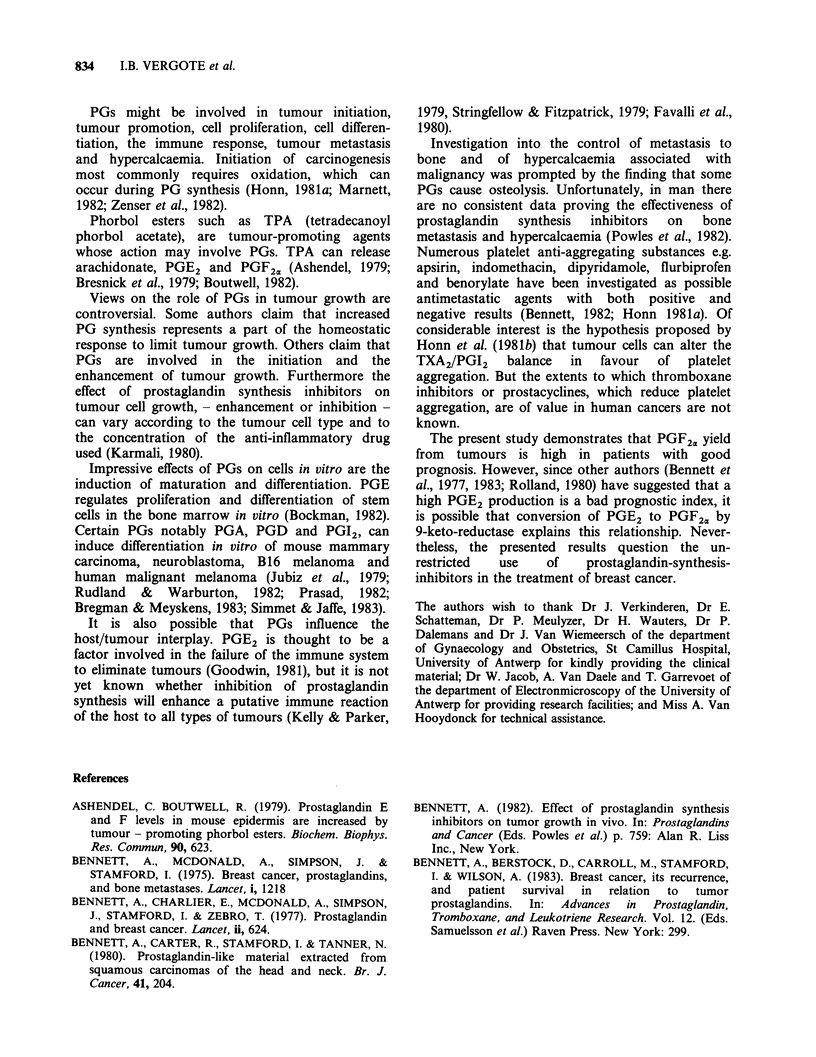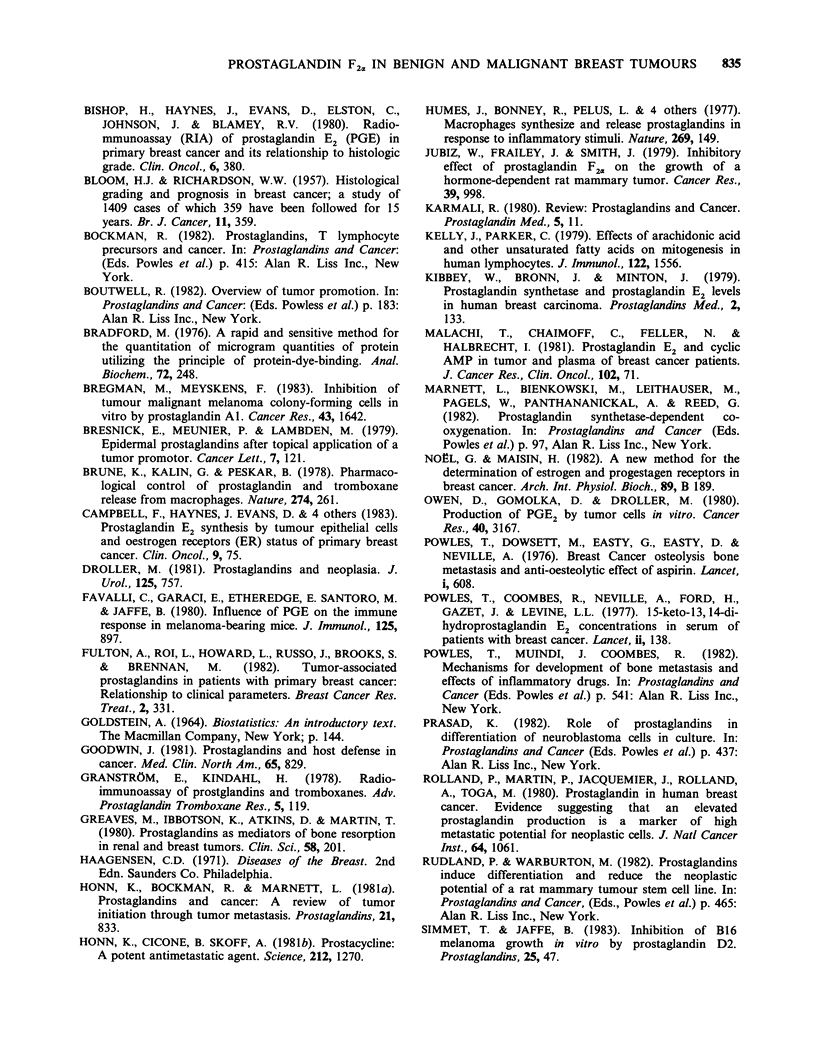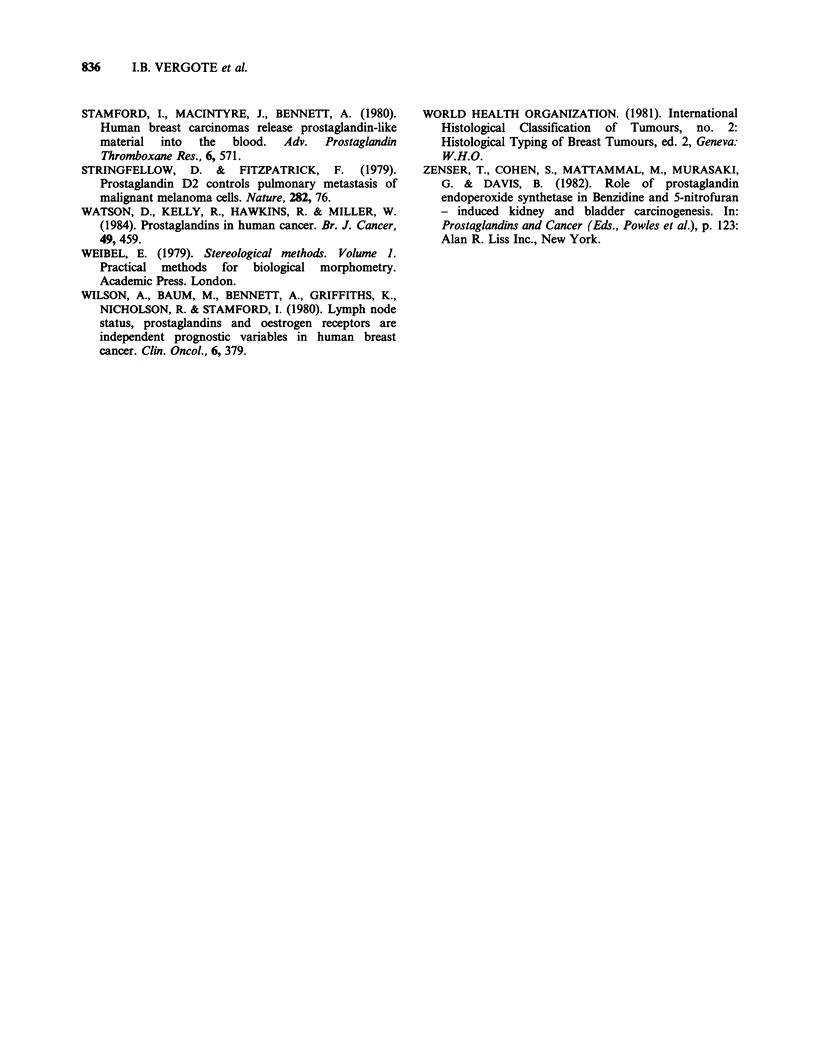# Prostaglandin F2 alpha in benign and malignant breast tumours.

**DOI:** 10.1038/bjc.1985.128

**Published:** 1985-06

**Authors:** I. B. Vergote, G. M. Laekeman, G. H. Keersmaekers, F. L. Uyttenbroeck, J. S. Vanderheyden, G. P. Albertyn, C. F. Haensch, G. J. De Roy, A. G. Herman

## Abstract

Prostaglandin F2 alpha (PGF2 alpha) was determined by radioimmunoassay in 57 breast carcinomata, 16 fibroadenomata, and 33 sclero-cystic-disease (SCD) specimens. In 41 cases of carcinoma and 10 cases of fibroadenoma, histologically non-malignant tissue was also obtained from the same breast. PGF2 alpha levels were significantly elevated in breast cancer when compared with the normal tissues and benign diseases (P less than 0.005 for each group). High PGF2 alpha levels were positively correlated with differentiation, positive oestrogen and progestagen receptor status, and low mitotic index. Tumours with good prognosis (less than 20 mm, negative lymph nodes, some degree of differentiation) showed significantly higher PGF2 alpha levels than tumours with a bad prognosis (greater than 20 mm, positive nodes and undifferentiated). A tendency for elevated PGF2 alpha levels was observed with negative lymphatic permeation, postmenopausal status, low grade of nuclear and cellular polymorphism and high degree of elastosis and fibrosis. No correlation was observed between PGF2 alpha levels and host-cell reaction. Plasma levels of 15-keto-13, 14-dihydro-PGF2 alpha were not elevated in cancer patients when compared with the SCD-group. The present study demonstrates that PGF2 alpha levels are high in tumours with good prognosis. However, since other authors have suggested that a high PGE2 production is a bad prognostic index, it is possible that conversion of PGE2 to PGF2 alpha by 9-keto-reductase explains this relationship. Nevertheless, the presented results question the unrestricted use of prostaglandin-synthesis-inhibitors in the treatment of breast cancer.


*Br. J. Cancer* (1985), **51**, 827–836

### Prostaglandin $F_{2\alpha}$ in benign and malignant breast tumours

I.B. Vergote[1], G.M. Laekeman[2], G.H. Keersmaekers[1], F.L. Uyttenbroeck[1], J.S. Vanderheyden[1], G.P. Albertyn[1], C.F. Haensch[1], G.J. De Roy[3] & A.G. Herman[2]

[1]*Departments of Gynaecology and Obstetrics, and Pathology, St. Camillus Hospital, Antwerp;* [2]*Department of Pharmacology, University of Antwerp; and* [3]*Department of Pathology, Brugmann Hospital, University of Brussels, Belgium*

**Summary** Prostaglandin $F_{2\alpha}$ ($PGF_{2\alpha}$) was determined by radioimmunoassay in 57 breast carcinomata, 16 fibroadenomata, and 33 sclero-cystic-disease (SCD) specimens. In 41 cases of carcinoma and 10 cases of fibroadenoma, histologically non-malignant tissue was also obtained from the same breast. $PGF_{2\alpha}$ levels were significantly elevated in breast cancer when compared with the normal tissues and benign diseases ($P<0.005$ for each group). High $PGF_{2\alpha}$ levels were positively correlated with differentiation, positive oestrogen and progestagen receptor status, and low mitotic index. Tumours with good prognosis (<20 mm, negative lymph nodes, some degree of differentiation) showed significantly higher $PGF_{2\alpha}$ levels than tumours with a bad prognosis(>20 mm, positive nodes and undifferentiated). A tendency for elevated $PGF_{2\alpha}$ levels was observed with negative lymphatic permeation, postmenopausal status, low grade of nuclear and cellular polymorphism and high degree of elastosis and fibrosis. No correlation was observed between $PGF_{2\alpha}$ levels and host-cell reaction.

Plasma levels of 15-keto-13,14-dihydro-$PGF_{2\alpha}$ were not elevated in cancer patients when compared with the SCD-group.

The present study demonstrates that $PGF_{2\alpha}$ levels are high in tumours with good prognosis. However, since other authors have suggested that a high $PGE_2$ production is a bad prognostic index, it is possible that conversion of $PGE_2$ to $PGF_{2\alpha}$ by 9-keto-reductase explains this relationship. Nevertheless, the presented results question the unrestricted use of prostaglandin-synthesis-inhibitors in the treatment of breast cancer.

Prostaglandins (PGs), especially of the E-series, have been shown to be elevated in a large number of human and experimental tumours. Special efforts have been made to investigate the features and the role of PG-synthesis in human breast cancer (Bennett *et al.*, 1975; Powles *et al.*, 1976; Kibbey *et al.*, 1980; Greaves *et al.*, 1980; Malachi *et al.*, 1981; Campbell *et al.*, 1983).

A considerable volume of research on the mechanisms of action of PGs in a wide variety of cells and tissues, indicates that PGs are possibly involved in tumour initiation, tumour promotion, cell proliferation and differentiation, the immune response, tumour metastasis, osteolysis and hypercalcaemia (Karmali, 1980; Honn *et al.*, 1981*a*; Droller, 1981; Goodwin, 1981).

The present study was designed to describe the basic features of $PGF_{2\alpha}$ production in benign and malignant breast tumours. Normal glandular breast tissues were used as controls. We determined $PGF_{2\alpha}$ because this product is more stable than $PGE_2$ and because prostaglandin $F_{2\alpha}$ has not been thoroughly investigated in human mammary cancer.

In order to find out whether the high levels of prostaglandin $F_{2\alpha}$ found in cancerous tissues could be correlated with either metastatic potential or with other prognostic unfavourable variables, the $PGF_{2\alpha}$ levels were examined in relation to the size of the tumour, axillary lymph node status, lymphatic vessel permeation, histological type and differentiation of the tumour, mitotix index, oestrogen and progestagen receptor status, and age and menopausal status of the patient.

Tumour – associated host-cells can produce considerable amounts of prostaglandins (Humes *et al.*, 1977; Brune *et al.*, 1978). Therefore the numbers of host-derived cells and the amount of necrosis were evaluated by means of quantitative microscopy. The epithelial cellularity was also evaluated by morphometric determination of the mean nuclear density and mean nuclear area.

### Materials and methods

There were 165 specimens from 106 patients who underwent surgery for a breast lump. Each

Correspondence: I.B. Vergote The Norwegian Radium Hospital, Department of Gynecologic Oncology. Montebello, Oslo 3, Norway.

Received 14 August 1984; and in revised form 6 February 1985.

© The Macmillan Press Ltd., 1985

specimen was divided into two representative parts and immediately immersed, either in acetone cooled by solid $CO_2$ ($-70$°C) for PG-investigations, or in Bouin's liquid for histopathological examination.

The tissue samples for $PGF_{2\alpha}$ investigation were then stored at $-30$°C until radioimmunoassay was performed. Sections 8 $\mu$m thick were cut from the stored routine embedded blocks, processed by standard methods, and stained by haematoxylin and eosin.

Fifty-seven tumours were diagnosed as primary breast cancer (patient age range: 31 to 80 y; mean: 56.6 y). No patient had received chemotherapy or radiotherapy at the time of biopsy. The patients were classified according to the pathological TNM system (UICC, Livre de Poche): $pT_{1a}N_0$16; $pT_{1a}N_{1a}$1; $pT_{1a}N_{1b}$6; $pT_{1a}N_2$3; $pT_{1b}N_0$1; $pT_{2a}N_0$11; $pT_{2a}N_{1a}$1; $pT_{2a}N_{1b}$10; $pT_{2a}N_2$2; $pT_{3a}N_{1b}$5 and $pT_4N_{1b}$1. Forty-nine tumours were benign, including 16 fibroadenomata (age range: 17 to 52 y; mean: 29.1 y), and 33 sclero-cystoc-disease specimens (age range: 30 to 62 y; mean: 46.2 y). The $PGF_{2\alpha}$ levels were also investigated in two cellular intracanalicular fibroadenomata.

Histologically proven normal breast tissue from 41 breasts with a carcinoma, and 10 from breasts with an adenofibroma, were investigated. In addition 6 lymph nodes, two of which showed metastasis, were examined.

In blood removed at the time of surgery the serum level of 15-keto-13,14-dihydro-$PGF_{2\alpha}$, the main metabolite of $PGF_{2\alpha}$, was measured in 11 cancer patients and in 16 patients with sclerocystic-disease. The estimation of the PGF metabolite rather than $PGF_{2\alpha}$ as such was preferred, since the former more closely reflects the true production *in vivo* and is less liable to erroneous changes during blood sampling.

The age and menopausal status of the patients and the tumour size at anatomopathological examination were recorded. Menopause was defined as at least one year after the last menstrual period.

Twenty-one cancerous lesions were examined for oestrogen and progestagen receptor status, determined according to Noël *et al.* (1982). In our laboratory, the oestrogen receptor status and progestagen receptor status are considered positive for levels higher than 10 and 100 fmole mg$^{-1}$ protein respectively.

The $PGF_{2\alpha}$ amounts were expressed as ng $PGF_{2\alpha}$ mg$^{-1}$ protein and as ng $PGF_{2\alpha}$ per cellularity index (g wet w tissue divided by the mean nuclear density). The number of evaluated samples is higher when the cellularity index is used, as there was not always enough tissue to measure the protein content. Also, the histological variables could not always be measured.

*Histopathology*

The slides were independently reviewed by three of the authors and were re-evaluated by a senior pathologist in cases of discordance. The results of the PG investigation were not known at that time.

Histological type, differentiation, infiltration, fibrosis, elastosis, host-cell reaction, lymph node metastasis, lymphatic vessel invasion, nuclear and cellular polymorphism and nuclear: cytoplasmic ratio, were independently evaluated.

Tumours were classified according to the WHO classification (1981). Whenever there was a combination of more than one histological type, the tumour was assigned to the group to which its most extensive component belonged. Tumours with a high amount of intraductal necrosis were classified as comedocarcinomata (Haagensen, 1971). The malignant tumours were graded according to Bloom & Richardson (1957).

Inflitration, fibrosis, elastosis and host-cell reaction were determined as negative, mild, moderate or strong. Nuclear and cellular polymorphism were calssified as mild, moderate or strong. The nuclear: cytoplasmic ratio was recorded as small, moderate or high.

Axillary lymph node status was recorded positive or negative depending on whether cancerous cells were present or not. Lymphatic vessel permeation was recorded as positive when cancerous cells were present in the lymphatic vessels in the tumour, or when the lymph node status was positive.

*Quantitative microscopy*

Nuclear density of the carcinomatous cells, nuclear size, mitotic index and necrosis, were evaluated morphometrically, according to Weibel (1979), using a planimeter (Kontron – MOP AM 02) and a microprocessor (HP 98 15 A).

The necrosis and total area of all the carcinomatas were determined with semi-automatic histomorphometry. Presence of necrosis was evaluated as percentage of the total area of representative tumour tissue.

Mean nuclear density and mean nuclear area of the malignant epithelial cells were counted on a projection microscope at ×400 magnification using the planimeter and microprocessor. Twenty areas of 6680 $\mu m^2$ were counted according to Weibel (1979).

The numbers of mitoses were counted in 20 random fields at ×400 magnification. Once focussed, no further adjustment was allowed and the structures that could be differently interpreted were not counted. The mitotic index was expressed as the mean of the number of mitoses counted in the 20 evaluated fields.

*Radioimmunoassay of $PGF_{2\alpha}$*

Before radioimmunoassay of $PGF_{2\alpha}$ the acetone was evaporated under nitrogen and the weight of the tissue determined. Tris buffer (50 $\mu$M, pH = 8.00 at 25°C) was added (3 ml $g^{-1}$ tissue) and sonicated for 90 min (Bransonic). Ice was regularly added to the bath fluid of the sonication apparatus to keep temperature below 10°C. The supernatant was separated from the tissue after centrifugation at 10,000 *g* (Eppendorf centrifuge). The extraction yield for PG was checked by adding trace amounts of radiolabelled standards.

Incubation of thawed and $N_2$-dried tissues (30 min, 37°C) with radioactive $^{14}C$ arachidonic acid ($n = 5$) showed almost complete inactivation of cyclo-oxygenase after acetone (−70°C) treatment since 95–98% of the substrate remained intact.

Samples (0.2 ml and 0.1 ml) were run in an adapted radioimmunoassay according to Granström & Kindahl (1978), using an antiserum which we produced in a final dilution of 1/17500. Cross reactivities at 50% of the binding curve were: $PGF_{1\alpha}$, 6.9%; 6-oxo-$PGF_{1\alpha}$, TXB2, $PGE_2$ and 15-keto-13, 14-di-hydro-$PGF_2{}^{\alpha}$, <0.001%.

A precipitate was formed with bovine-$\gamma$-globulin after adding polyethyleneglycol (PEG) 4000. Radioactivity was counted in a Packard 460 scintillation counter with quench correction. The protein content of the breast tissue extracts was determined (Bradford, 1976) and the $PGF_{2\alpha}$ content expressed as ng PG $mg^{-1}$ protein.

During surgery citrate plasma was collected from some patients with SCD or breast cancer. The plasma (0.5 ml and 0.2 ml) was analysed (RIA) for 15-keto-13, 14-dihydro-$PGF_{2\alpha}$, using the antiserum which we produced, in a final dilution of 1/7000. The cross-reactivities at 50% of the binding curve were: 6, 15-diketo-13, 14-dihydro-$PGF_{2\alpha}$ 21%; 6, 15-diketo-$PGF_{1\alpha}$, 3.3%; $PGF_{1\alpha}$, 0.2%; $PGF_{2\alpha}$, 0.04%; 6-keto-$PGF_{1\alpha}$, 0.03%; $PGE_2$, $TXB_2$ < 0.001%.

The extraction yield of $PGF_{2\alpha}$ from the specimens was 80.8 ± 4.1% ($n = 6$), calculated by adding trace amounts of radioactive $PGF_{2\alpha}$ to the tissues.

*Reagents*

*$PGF_{2\alpha}$* and 15-keto-13, 14-dihydro prostaglandin $F_{2\alpha}$ (Upjohn), [$^3$H]-radiolabelled $PGF_{2\alpha}$(NEN), [$^3$H]-radiolabelled 15-keto-13, 14-dihyrdo-$PGF_{2\alpha}$, trizma base and trizma HCl (Sigma), polyethyleneglycol 4000 (Purna), Instagel (Packard), bovine-$\gamma$-globulin (Sigma).

*Statistical analysis*

The used statistical test were: Wilcoxon (*u* value), one way analysis of variance (ANOVA; F value) and significance of the correlation coefficient (linear regression; *t* value) (Goldstein, 1964). When no statistical test is mentioned, the Wilcoxon test was used to compare two groups of samples.

### Results

*$PGF_{2\alpha}$ levels in relation to the histopathological groups*

The results according to the different histopathological groups are shown in Figure 1 and are analysed statistically in Table I. The $PGF_{2\alpha}$ yield from malignant tissue (CA) was higher than from non-malignant tissues from breast with a carcinoma

**Figure 1** $PGF_{2\alpha}$ levels extracted from biopsies of normal and pathological breast tissue. CA = malignant tumours ($n = 57$). N–CA = apparently normal tissue from a breast with a carcinoma ($n = 41$). SCD = sclero-cystic-disease tissues ($n = 33$). FA = fibradenomata ($n = 16$). N = normal breast tissue ($n = 10$). Median values are indicated.

**Table I** Comparison of the $PGF_{2\alpha}$ yield between the various histopathological groups.

| *Group* | $PGF_{2\alpha}$ | *Group* | $PGF_{2\alpha}$ | *P-value* |
|---|---|---|---|---|
| CA (57) | 15.00±2.35 | N–CA (41) | 0.95±0.27 | <0.005 |
| | | FA (16) | 2.20±0.57 | <0.005 |
| | | SCD (33) | 2.33±0.92 | <0.005 |
| | | N (10) | 0.66±0.21 | <0.005 |
| N (10) | 0.66±0.21 | FA (16) | 2.20±0.57 | <0.005 |
| | | N–CA (41) | 0.95±0.27 | 0.44 |
| | | SCD (33) | 2.33±0.92 | 0.10 |

The PG results are given as mean±s.e., and expressed as $ng\,mg^{-1}$ protein. The number of evaluated samples is given in brackets. CA: carcinoma N–CA: histologically non-malignant tissue from a breast with a carcinoma. FA: fibroadenoma. N: normal glandular breast tissue. SCD: sclerocystic disease.

(N-CA), fibroadenomata (FA), sclero-cystic-disease tissues (SCD) and normal glandular breast tissues (N) (all $P<0.0003$).

Amounts from fibroadenomata (FA) were higher than from normal glandular breast tissues (N) ($P=0.002$). We found no difference in the $PGF_{2\alpha}$ yields from the N and N–CA groups ($P=0.43$). $PGF_{2\alpha}$ yield tended to be higher from SCD specimens than from N specimens ($P=0.069$).

In 12 out of 26 patients a measurable 15-keto-13,14-dihydro-$PGF_{2\alpha}$ level was found, i.e. $>25\,pg\,ml^{-1}$. Only 3 patients showed plasma levels $>100\,pg\,ml^{-1}$ (475, 456 and $1065\,pg\,ml^{-1}$). We found no correlation between plasma levels of 15-keto-13,14-dihydro-$PGF_{2\alpha}$ and $PGF_{2\alpha}$ breast tissue levels. There was no apparent difference between the plasma levels found in the CA group and the SCD group.

### *Relationship between the breast cancer $PGF_{2\alpha}$ levels and the different prognostical variables*

*Tumour oestrogen and progestagen receptor contents* Oestrogen-positive tumours yielded more $PGF_{2\alpha}\,mg^{-1}$ protein, than did oestrogen-negative tumours ($P=0.004$); progesterone-positive tumours yielded more $PGF_{2\alpha}\,mg^{-1}$ protein ($P=0.05$) and per cellularity index ($P=0.037$) (Table II and Figure 2).

*Tumour size* $PGF_{2\alpha}$ levels tended to correlate inversely with the tumour size ($r=0.106$, $P=0.33$) (Table III).

*Age and menopausal status* Tumour $PGF_{2\alpha}$ levels $mg^{-1}$ protein tended to be higher with older patients (>60 years) (ANOVA; $F=2.70$; $P=0.06$), and with the cellularity index the $P$ value was 0.03 (ANOVA; $F=3.33$).

There was no significant difference between tumour $PGF_{2\alpha}$ levels of pre- and postmenopausal patients ($P=0.29$) (Table III).

*Histological type and differentiation* Statistical analysis of all the histological types was difficult, because the infiltrating ductal carcinomatas composed the only substantial group. They yielded more $PGF_{2\alpha}$ than did the other histological types (Table IV). The two cellular intracanalicular fibroadenomata yielded 0.2 and 4.0 ng $PGF_{2\alpha}\,mg^{-1}$ protein respectively.

Histological differentiation was recorded as small, moderate or high. For statistical analysis we divided

**Table II** $PGF_{2\alpha}$ yields from breast cancers in relation to steroid receptors and differentiation.

| *Variable* | | $PGF_{2\alpha}$ | *P-value* |
|---|---|---|---|
| Oestrogen | pos(12) | 15.04±2.96 | 0.004 |
| receptors | neg (9) | 5.54±1.07 | |
| Progesterone | pos(15) | 12.78±2.62 | 0.05 |
| receptors | neg (6) | 6.43±1.72 | |
| Differentiation | undiff(28) | 8.06±1.40 | 0.004 |
| | diff(24) | 14.80±2.14 | |

The PG results are given as $ng\,mg^{-1}$ protein, mean±s.e.
The number of evalulated samples is given in brackets.
Oestrogen receptor pos=oestrogen receptor contents≥ $10\,fmol\,mg^{-1}$ protein
Oestrogen receptor neg=oestrogen receptor content< $10\,fmol\,mg^{-1}$ protein
Proesterone receptor pos=progesterone receptor content ≥$100\,fmol\,mg^{-1}$ protein
Progesterone receptor neg=progesterone receptor content<$100\,fmol\,mg^{-1}$ protein
Differentiation undiff=undifferentiated
diff=some degree of differentiation (small, moderate or high).

**Figure 2** Relationship between progestagen and oestrogen receptors and $PGF_{2\alpha}$ levels. The PG results are given as mean ± s.e. The figures at the bottom represent the number of patients evaluated. (□) Progesterone receptors (−) = ≦100 fmol mg$^{-1}$ protein. (+) = >100 fmol mg$^{-1}$ protein. (▒) Oestrogen receptors (−) = ≦10 fmol mg$^{-1}$ protein. (+) = >10 fmol mg$^{-1}$ protein. ng $PGF_{2\alpha}$/cellularity index = ng $PGF_{2\alpha}$g$^{-1}$ wet tissue w/mean nuclear density. (*:$P$ = or <0.05; **$P$<0.005; When no * is indicated: $P$>0.05).

the tumours into two groups: undifferentiated and some degree of differentiation (small, moderate or high). The tumours with some degree of differentiation yielded more $PGF_{2\alpha}$ than did the undifferentiated tumours ($P$ = 0.004, ng mg$^{-1}$ protein; $P$ = 0.013, ng cellularity index$^{-1}$) (Table II and Figure 3). ANOVA between the various groups with some degree of differentiation showed no significant differences (F = 0.16, $P$ = 0.85).

*Lymph node metastasis and lymphatic vessel permeation* We found, at most, a weak tendency for higher $PGF_{2\alpha}$ levels in the lymph node negative group compared with the node positive group ($P$ = 0.189) (Table III). $PGF_{2\alpha}$ was determined in 6 lymph nodes, the values for the negative lymph nodes being 0.1, 7.7, 1.9 and 9.2 ng mg$^{-1}$ protein; those for the positive lymph nodes were 3.3 and 9.7 ng mg$^{-1}$ protein.

Lymphatic vessel permeation was present in 82% of the cases and there was little or no difference ($P$ = 0.209) between this group and the small negative group (Table III).

*Size and density of nuclei of carcinoma cells* We observed no correlation between the $PGF_{2\alpha}$ levels and the mean nuclear density or size ($P$ = 0.32; $r$ = −0.064 and $P$ = 0.49; $r$ = −0.002 respectively). We observed also no correlation with the mean nuclear area ($P$ = 0.30; $r$ = 0.076).

*Mitotic index* The mitotic index correlated inversely with the ng $PGF_{2\alpha}$ mg$^{-1}$ protein ($P$ = 0.05; $r$ = −0.227), but when the cellularity index was used, the significance was $P$ = 0.13 ($r$ = −0.159).

**Table III** $PGF_{2\alpha}$ yields from primary breast cancer in relation to several variables

| *Variable* | *$PGF_{2\alpha}$ yield* |
|---|---|
| Tumour size < 20 mm (25) | 11.72 ± 2.42 |
| 20– < 39 mm (21) | 10.91 ± 1.72 |
| ≧40 mm (7) | 9.31 ± 2.76 |
| Menopausal status pre (21) | 9.89 ± 1.45 |
| post (32) | 11.93 ± 1.81 |
| Lymph nodes negative (25) | 17.18 ± 4.15 |
| positive (28) | 12.26 ± 2.65 |
| Lymphatic permeation negative (7) | 11.90 ± 2.93 |
| positive (46) | 9.91 ± 1.17 |
| Nuclear and cellular polymorphism | |
| mild (7) | 14.20 ± 4.47 |
| moderate (33) | 10.55 ± 1.61 |
| strong (13) | 9.48 ± 1.93 |
| Nuclear cytoplasmic ratio: | |
| small (10) | 10.55 ± 2.44 |
| moderate (34) | 11.91 ± 1.74 |
| high (9) | 7.07 ± 1.45 |
| [a]Host cell reaction: | |
| mild (28) | 13.67 ± 1.88 |
| moderate (12) | 9.07 ± 2.59 |
| strong (8) | 7.59 ± 1.33 |
| [a]Mast cells: | |
| absent (11) | 8.50 ± 1.57 |
| present (17) | 12.40 ± 2.64 |
| Necrosis 0% (9) | 10.77 ± 3.62 |
| >0% (25) | 10.24 ± 1.84 |
| ≧1% (19) | 11.38 ± 1.95 |
| Fibrosis: | |
| mild (17) | 9.54 ± 1.90 |
| moderate (25) | 10.53 ± 1.89 |
| marked (11) | 12.66 ± 3.20 |
| Elastosis: | |
| 0 (32) | 8.35 ± 0.97 |
| mild (14) | 14.37 ± 3.56 |
| moderate (5) | 18.62 ± 3.69 |
| marked (2) | 2.7 and 10.0 |
| Infiltration: | |
| 0 (1) | 21 |
| mild (0) | — |
| moderate (12) | 7.53 ± 1.35 |
| strong (39) | 12.01 ± 1.55 |
| marked (1) | 4.5 |

The PG results are given as ng mg$^{-1}$ protein, mean ± s.e.m. The number of evaluated samples is given in brackets. [a]Cases difficult to classify were not evalulated.

**Table IV** $PGF_{2\alpha}$ yields from breast cancer in relation to histological type

| *Histological type* | $PGF_{2\alpha}$ |
|---|---|
| Infiltrating ductal undiff (19) | 9.72±1.92 |
| diff (24) | 14.80±2.14 |
| Lobular (5) | 3.38±0.44 |
| Comedo (3) | 2.3 to 11.2[a] |
| Medullary (1) | 6.0 |
| Mucoid (1) | 4.5 |

The PG results are given as $ng\,mg^{-1}$ protein, mean±s.e.m. The number of evalulated samples is given in brackets.

Undiff = undifferentiated.

Diff = some degree of differentiation (small, moderate or high).

[a] = limit values.

**Figure 3** Relationship between the degree of differentiation and $PGF_{2\alpha}$ levels. The PG results are given as mean±s.e. The number of patients evaluated is indicated at the bottom. − = undifferentiated; + to +++ = some degree of differentiation (mild, moderate or high). ng $PGF_{2\alpha}$/cellularity index = ng $PGF_{2\alpha}\,g^{-1}$ wet tissue w/mean nuclear denisty. (*$P<0.05$).

*Nuclear and cellular polymorphism and the nuclear: cytoplasmic ratio* Tumours with a low degree of nuclear and cellular polymorphism and a low nuclear: cytoplasmic ratio showed at most a weak tendency to yield more $PGF_{2\alpha}$ (ANOVA, $F=0.60$; $P=0.55$; and $F=1.64$, $P=0.20$ respectively) (Table III).

*Host cell reaction and necrosis* PG levels tended to correlate inversely with the host-cell reaction (ANOVA, $F=2.01$, $P=0.15$), but there was little or no relationship to the presence of mast cells ($P=0.37$), or to the amount of necrosis (ANOVA, $F=0.13$; $P=0.88$) (Table III).

*Elastosis, fibrosis and infiltration* $PGF_{2\alpha}$ values in tumours with elastosis, fibrosis or infiltration showed at most a week tendency to be higher (Wilcoxon, $P=0.10$; ANOVA, $F=0.67$, $P=0.52$; Wilcoxon, $P=0.27$ respectively).

*Cumulation of variables* A gradual increase in statistical significance occurred when several characteristics were combined. Mean $PGF_{2\alpha}$ levels $ng\,mg^{-1}$ protein tended to be higher when the lymph nodes were negative ($P=0.189$), and the relationship marginally strengthens when tumours <20 mm with negative lymp nodes are compared with tumours >20 mm with positive lymph nodes ($P=0.104$). Tumours with some degree of differentiation <20 mm and with negative lymph nodes yielded more $PGF_{2\alpha}$ than did undifferentiated tumours >20 mm with positive lymph nodes ($P=0.045$) (Table V).

### Discussion

The present study has examined $PGF_{2\alpha}$ tissue levels in benign and malignant breast tumours. This is to our knowledge the first study which has determined by radioimmunoassay the $PGF_{2\alpha}$ tissue levels in breast cancers and compared them with histologically proven non-malignant tissues from the same breast with correlations in terms of $PGF_{2\alpha}\,mg^{-1}$ protein and cellularity.

Bennett *et al.* (1975, 1977) using bioassay first described elevated levels of "prostaglandin-like' material in extracts of human mammary cancer. Greaves *et al.* (1980) performed radioiommunoassay of PGE and PGF and correlated the results with tissue weight in a small group of breast cancers ($n=16$). Rolland *et al.* (1980) determined the $PGE_2$ production from added arachidonic acid in 91 breast cancers. Bishop *et al.* (1980) and Malachi *et al.* (1981) determined $PGE_2$ and Fulton et al. (1982) $PGE_2$and $PGF_{2\alpha}$ by radioimmunoassay in breast cancer and correlated the results with tissue wet w. Watson *et al.* (1984) measured $PGE_2$ and $PGF_{2\alpha}$ by Gas Liquid Chromatography – Mass Spectrometry in 100 mammary carcinomatas.

Our difference between the $PGF_{2\alpha}\,mg^{-1}$ protein levels of cancer tissue and histologically proven

**Table V** $PGF_{2\alpha}$ yields from breast cancer in relation to cumulated prognostical factors.

| *Variable* | $PGF_{2\alpha}$ | *P-value* |
|---|---|---|
| Lymph nodes pos (28) | $12.26 \pm 2.65$ | 0.190 |
| neg (25) | $17.18 \pm 4.15$ | |
| Lymph nodes pos + tumour size > 20 mm (19) | $12.88 \pm 3.65$ | 0.104 |
| neg + tumour size ≦ 20 mm (16) | $18.97 \pm 5.67$ | |
| Lymph nodes pos + tumour size > 20 mm + undiff (12) | $13.60 \pm 5.58$ | 0.045 |
| neg + tumour size ≦ 20 mm + diff (8) | $22.95 \pm 9.40$ | |

The PG results are given as ng mg$^{-1}$ protein, mean ± s.e.m.
The number of evalulated samples is given in brackets.
Undiff = undifferentiated.
Diff = differentiated: some degree of differentiation (small, moderate or high).

non-malignant tissue of the same breast confirms the findings in the studies cited above.

We also found statistically significant different $PGF_{2\alpha}$ yields from cancers, compared with fibro-adenomata, sclero-cystic-disease and normal glandular breast tissue (Table I).

Stamford *et al.* (1980) showed an increase of prostaglandin-like material in extracts of blood draining breast carcinomatas. In the study of Powles *et al.* (1977) 15-keto-13, 14-dihydro-$PGE_2$ was elevated, especially in the group of breast cancer patients who had metastasis. In the study of Malachi *et al.* (1981) the plasma $PGE_2$-metabolite concentration did not reflect the $PGE_2$ tissue levels, and no difference was found between the benign and malignant cases, but both concentrations were higher than those of the healthy controls. In this study none of the patients had overt metastasis at the time of biopsy. We could not demonstrate a higher level of 15-keto-13, 14-dihydro-$PGF_{2\alpha}$ in the plasma of the cancer patients than in the SCD group, or a correlation between the plasma levels of the $PGF_{2\alpha}$-metabolite and the $PGF_{2\alpha}$ tissue levels.

Bennett *et al.* (1977, 1983) showed that the ability of malignant breast tumours to produce prostaglandin-like material correlates inversely with patient survival. Rolland *et al.* (1980) concluded that a high $PGE_2$ production occurs very early in the development of a malignant tumour, and that an elevated prostaglandin production seems to be associated with metastasis. This relationship does not seem to exist for $PGF_{2\alpha}$ tissue levels.

In the present study high $PGF_{2\alpha}$ levels correlate with good prognostic variables (differentiation, positive oestrogen and progestagen receptor status, low mitotic index). Tumours with a good prognosis (some degree of differentiation, < 20 mm, no lymph node metastasis) yielded more $PGF_{2\alpha}$ than did tumours with a bad prognosis (undifferentiated, > 20 mm, lymph node metastasis) Table V), although the relationships with these uncombined variables were weak.

We also observed tendencies for higher $PGF_{2\alpha}$ levels with negative lymphatic permeation, post-menopausal patients, fibrosis, elastosis and a low nuclear: cytoplasmic ratio (Table III).

Malachi *et al.* (1981) found no correlation between $PGE_2$ tissue levels and survival, histological type and stage. The data on tumour recurrence of Bennett *et al.* (1983) argued against an important role for $PGE_2$ in bone metastasis, and Blamey (personal communication) did not find a correlation between $PGE_2$ and bone metastasis. In a series of rat mammary carcinomatas $PGE_2$ levels correlated inversely with metastatic potential (Kibbey *et al.*, 1979).

Fulton *et al.* (1982) and Campbell *et al.* (1983) concluded that oestrogen receptor – positive tumours synthesized more $PGE_2$, whereas Rolland *et al.* (1980) found a tendency for the opposite. Wilson *et al.* (1980) and Watson *et al.* (1984) found little or no correlation between prostaglandins and oestrogen receptor status. In the present study $PGF_{2\alpha}$ correlated with receptors for steroid hormones.

Host-derived inflammatory cells are thought to contribute to the PG-production by cancers (Greaves *et al.*, 1980; Honn *et al.*, 1981*a*). Bennett *et al.* (1980) correlated total "$PGE_2$-equivalents" with the extent of inflammation and necrosis in X-irradiated squamous carcinomata of head and neck. We found no significant relationship between the $PGF_{2\alpha}$ levels and the intensity of the host cell reaction, presence of mast cells or necrosis. This corresponds with the finding that most of the inflamatory cells were lymphocytes, and that co-incubation of carcinogen-induced rat bladder tumour cells with lymphocytes did not significantly change the $PGE_2$ yield (Owen *et al.*, 1980).

PGs might be involved in tumour initiation, tumour promotion, cell proliferation, cell differentiation, the immune response, tumour metastasis and hypercalcaemia. Initiation of carcinogenesis most commonly requires oxidation, which can occur during PG synthesis (Honn, 1981*a*; Marnett, 1982; Zenser *et al.*, 1982).

Phorbol esters such as TPA (tetradecanoyl phorbol acetate), are tumour-promoting agents whose action may involve PGs. TPA can release arachidonate, $PGE_2$ and $PGF_{2\alpha}$ (Ashendel, 1979; Bresnick *et al.*, 1979; Boutwell, 1982).

Views on the role of PGs in tumour growth are controversial. Some authors claim that increased PG synthesis represents a part of the homeostatic response to limit tumour growth. Others claim that PGs are involved in the initiation and the enhancement of tumour growth. Furthermore the effect of prostaglandin synthesis inhibitors on tumour cell growth, – enhancement or inhibition – can vary according to the tumour cell type and to the concentration of the anti-inflammatory drug used (Karmali, 1980).

Impressive effects of PGs on cells *in vitro* are the induction of maturation and differentiation. PGE regulates proliferation and differentiation of stem cells in the bone marrow *in vitro* (Bockman, 1982). Certain PGs notably PGA, PGD and $PGI_2$, can induce differentiation *in vitro* of mouse mammary carcinoma, neuroblastoma, B16 melanoma and human malignant melanoma (Jubiz *et al.*, 1979; Rudland & Warburton, 1982; Prasad, 1982; Bregman & Meyskens, 1983; Simmet & Jaffe, 1983).

It is also possible that PGs influence the host/tumour interplay. $PGE_2$ is thought to be a factor involved in the failure of the immune system to eliminate tumours (Goodwin, 1981), but it is not yet known whether inhibition of prostaglandin synthesis will enhance a putative immune reaction of the host to all types of tumours (Kelly & Parker, 1979, Stringfellow & Fitzpatrick, 1979; Favalli *et al.*, 1980).

Investigation into the control of metastasis to bone and of hypercalcaemia associated with malignancy was prompted by the finding that some PGs cause osteolysis. Unfortunately, in man there are no consistent data proving the effectiveness of prostaglandin synthesis inhibitors on bone metastasis and hypercalcaemia (Powles *et al.*, 1982). Numerous platelet anti-aggregating substances e.g. apsirin, indomethacin, dipyridamole, flurbiprofen and benorylate have been investigated as possible antimetastatic agents with both positive and negative results (Bennett, 1982; Honn 1981*a*). Of considerable interest is the hypothesis proposed by Honn *et al.* (1981*b*) that tumour cells can alter the $TXA_2/PGI_2$ balance in favour of platelet aggregation. But the extents to which thromboxane inhibitors or prostacyclines, which reduce platelet aggregation, are of value in human cancers are not known.

The present study demonstrates that $PGF_{2\alpha}$ yield from tumours is high in patients with good prognosis. However, since other authors (Bennett *et al.*, 1977, 1983; Rolland, 1980) have suggested that a high $PGE_2$ production is a bad prognostic index, it is possible that conversion of $PGE_2$ to $PGF_{2\alpha}$ by 9-keto-reductase explains this relationship. Nevertheless, the presented results question the unrestricted use of prostaglandin-synthesis-inhibitors in the treatment of breast cancer.

The authors wish to thank Dr J. Verkinderen, Dr E. Schatteman, Dr P. Meulyzer, Dr H. Wauters, Dr P. Dalemans and Dr J. Van Wiemeersch of the department of Gynaecology and Obstetrics, St Camillus Hospital, University of Antwerp for kindly providing the clinical material; Dr W. Jacob, A. Van Daele and T. Garrevoet of the department of Electronmicroscopy of the University of Antwerp for providing research facilities; and Miss A. Van Hooydonck for technical assistance.

### References

ASHENDEL, C. BOUTWELL, R. (1979). Prostaglandin E and F levels in mouse epidermis are increased by tumour – promoting phorbol esters. *Biochem. Biophys. Res. Commun*, **90**, 623.

BENNETT, A., MCDONALD, A., SIMPSON, J. & STAMFORD, I. (1975). Breast cancer, prostaglandins, and bone metastases. *Lancet*, **i**, 1218

BENNETT, A., CHARLIER, E., MCDONALD, A., SIMPSON, J., STAMFORD, I. & ZEBRO, T. (1977). Prostaglandin and breast cancer. *Lancet*, **ii**, 624.

BENNETT, A., CARTER, R., STAMFORD, I. & TANNER, N. (1980). Prostaglandin-like material extracted from squamous carcinomas of the head and neck. *Br. J. Cancer*, **41**, 204.

BENNETT, A. (1982). Effect of prostaglandin synthesis inhibitors on tumor growth in vivo. In: *Prostaglandins and Cancer* (Eds. Powles *et al.*) p. 759: Alan R. Liss Inc., New York.

BENNETT, A., BERSTOCK, D., CARROLL, M., STAMFORD, I. & WILSON, A. (1983). Breast cancer, its recurrence, and patient survival in relation to tumor prostaglandins. In: *Advances in Prostaglandin, Tromboxane, and Leukotriene Research*. Vol. 12. (Eds. Samuelsson *et al.*) Raven Press. New York: 299.

BISHOP, H., HAYNES, J., EVANS, D., ELSTON, C., JOHNSON, J. & BLAMEY, R.V. (1980). Radio-mmunoassay (RIA) of prostaglandin $E_2$ (PGE) in primary breast cancer and its relationship to histologic grade. *Clin. Oncol.*, **6**, 380.

BLOOM, H.J. & RICHARDSON, W.W. (1957). Histological grading and prognosis in breast cancer; a study of 1409 cases of which 359 have been followed for 15 years. *Br. J. Cancer*, **11**, 359.

BOCKMAN, R. (1982). Prostaglandins, T lymphocyte precursors and cancer. In: *Prostaglandins and Cancer*: (Eds. Powles *et al.*) p. 415: Alan R. Liss Inc., New York.

BOUTWELL, R. (1982). Overview of tumor promotion. In: *Prostaglandins and Cancer*: (Eds. Powless *et al.*) p. 183: Alan R. Liss Inc., New York.

BRADFORD, M. (1976). A rapid and sensitive method for the quantitation of microgram quantities of protein utilizing the principle of protein-dye-binding. *Anal. Biochem.*, **72**, 248.

BREGMAN, M., MEYSKENS, F. (1983). Inhibition of tumour malignant melanoma colony-forming cells in vitro by prostaglandin A1. *Cancer Res.*, **43**, 1642.

BRESNICK, E., MEUNIER, P. & LAMBDEN, M. (1979). Epidermal prostaglandins after topical application of a tumor promotor. *Cancer Lett.*, **7**, 121.

BRUNE, K., KALIN, G. & PESKAR, B. (1978). Pharmacological control of prostaglandin and tromboxane release from macrophages. *Nature*, **274**, 261.

CAMPBELL, F., HAYNES, J. EVANS, D. & 4 others (1983). Prostaglandin $E_2$ synthesis by tumour epithelial cells and oestrogen receptors (ER) status of primary breast cancer. *Clin. Oncol.*, **9**, 75.

DROLLER, M. (1981). Prostaglandins and neoplasia. *J. Urol.*, **125**, 757.

FAVALLI, C., GARACI, E., ETHEREDGE, E. SANTORO, M. & JAFFE, B. (1980). Influence of PGE on the immune response in melanoma-bearing mice. *J. Immunol.*, **125**, 897.

FULTON, A., ROI, L., HOWARD, L., RUSSO, J., BROOKS, S. & BRENNAN, M. (1982). Tumor-associated prostaglandins in patients with primary breast cancer: Relationship to clinical parameters. *Breast Cancer Res. Treat.*, **2**, 331.

GOLDSTEIN, A. (1964). *Biostatistics: An introductory text.* The Macmillan Company, New York; p. 144.

GOODWIN, J. (1981). Prostaglandins and host defense in cancer. *Med. Clin. North Am.*, **65**, 829.

GRANSTRÖM, E., KINDAHL, H. (1978). Radioimmunoassay of prostglandins and tromboxanes. *Adv. Prostaglandin Tromboxane Res.*, **5**, 119.

GREAVES, M., IBBOTSON, K., ATKINS, D. & MARTIN, T. (1980). Prostaglandins as mediators of bone resorption in renal and breast tumors. *Clin. Sci.*, **58**, 201.

HAAGENSEN, C.D. (1971). *Diseases of the Breast*. 2nd Edn. Saunders Co. Philadelphia.

HONN, K., BOCKMAN, R. & MARNETT, L. (1981*a*). Prostaglandins and cancer: A review of tumor initiation through tumor metastasis. *Prostaglandins*, **21**, 833.

HONN, K., CICONE, B. SKOFF, A. (1981*b*). Prostacycline: A potent antimetastatic agent. *Science*, **212**, 1270.

HUMES, J., BONNEY, R., PELUS, L. & 4 others (1977). Macrophages synthesize and release prostaglandins in response to inflammatory stimuli. *Nature*, **269**, 149.

JUBIZ, W., FRAILEY, J. & SMITH, J. (1979). Inhibitory effect of prostaglandin $F_{2\alpha}$ on the growth of a hormone-dependent rat mammary tumor. *Cancer Res.*, **39**, 998.

KARMALI, R. (1980). Review: Prostaglandins and Cancer. *Prostaglandin Med.*, **5**, 11.

KELLY, J., PARKER, C. (1979). Effects of arachidonic acid and other unsaturated fatty acids on mitogenesis in human lymphocytes. *J. Immunol.*, **122**, 1556.

KIBBEY, W., BRONN, J. & MINTON, J. (1979). Prostaglandin synthetase and prostaglandin $E_2$ levels in human breast carcinoma. *Prostaglandins Med.*, **2**, 133.

MALACHI, T., CHAIMOFF, C., FELLER, N. & HALBRECHT, I. (1981). Prostaglandin $E_2$ and cyclic AMP in tumor and plasma of breast cancer patients. *J. Cancer Res., Clin. Oncol.*, **102**, 71.

MARNETT, L., BIENKOWSKI, M., LEITHAUSER, M., PAGELS, W., PANTHANANICKAL, A. & REED, G. (1982). Prostaglandin synthetase-dependent co-oxygenation. In: *Prostaglandins and Cancer* (Eds. Powles *et al.*) p. 97, Alan R. Liss Inc., New York.

NOËL, G. & MAISIN, H. (1982). A new method for the determination of estrogen and progestagen receptors in breast cancer. *Arch. Int. Physiol. Bioch.*, **89**, B 189.

OWEN, D., GOMOLKA, D. & DROLLER, M. (1980). Production of $PGE_2$ by tumor cells *in vitro*. *Cancer Res.*, **40**, 3167.

POWLES, T., DOWSETT, M., EASTY, G., EASTY, D. & NEVILLE, A. (1976). Breast Cancer osteolysis bone metastasis and anti-oesteolytic effect of aspirin. *Lancet*, **i**, 608.

POWLES, T., COOMBES, R., NEVILLE, A., FORD, H., GAZET, J. & LEVINE, L.L. (1977). 15-keto-13,14-dihydroprostaglandin $E_2$ concentrations in serum of patients with breast cancer. *Lancet*, **ii**, 138.

POWLES, T., MUINDI, J. COOMBES, R. (1982). Mechanisms for development of bone metastasis and effects of inflammatory drugs. In: *Prostaglandins and Cancer* (Eds. Powles *et al.*) p. 541: Alan R. Liss Inc., New York.

PRASAD, K. (1982). Role of prostaglandins in differentiation of neuroblastoma cells in culture. In: *Prostaglandins and Cancer* (Eds. Powles *et al.*) p. 437: Alan R. Liss Inc., New York.

ROLLAND, P., MARTIN, P., JACQUEMIER, J., ROLLAND, A., TOGA, M. (1980). Prostaglandin in human breast cancer. Evidence suggesting that an elevated prostaglandin production is a marker of high metastatic potential for neoplastic cells. *J. Natl Cancer Inst.*, **64**, 1061.

RUDLAND, P. & WARBURTON, M. (1982). Prostaglandins induce differentiation and reduce the neoplastic potential of a rat mammary tumour stem cell line. In: *Prostaglandins and Cancer*, (Eds., Powles *et al.*) p. 465: Alan R. Liss Inc., New York.

SIMMET, T. & JAFFE, B. (1983). Inhibition of B16 melanoma growth *in vitro* by prostaglandin D2. *Prostaglandins*, **25**, 47.

STAMFORD, I., MACINTYRE, J., BENNETT, A. (1980). Human breast carcinomas release prostaglandin-like material into the blood. *Adv. Prostaglandin Thromboxane Res.*, **6**, 571.

STRINGFELLOW, D. & FITZPATRICK, F. (1979). Prostaglandin D2 controls pulmonary metastasis of malignant melanoma cells. *Nature*, **282**, 76.

WATSON, D., KELLY, R., HAWKINS, R. & MILLER, W. (1984). Prostaglandins in human cancer. *Br. J. Cancer*, **49**, 459.

WEIBEL, E. (1979). *Stereological methods. Volume 1.* Practical methods for biological morphometry. Academic Press. London.

WILSON, A., BAUM, M., BENNETT, A., GRIFFITHS, K., NICHOLSON, R. & STAMFORD, I. (1980). Lymph node status, prostaglandins and oestrogen receptors are independent prognostic variables in human breast cancer. *Clin. Oncol.*, **6**, 379.

WORLD HEALTH ORGANIZATION. (1981). International Histological Classification of Tumours, no. 2: Histological Typing of Breast Tumours, ed. 2, *Geneva: W.H.O.*

ZENSER, T., COHEN, S., MATTAMMAL, M., MURASAKI, G. & DAVIS, B. (1982). Role of prostaglandin endoperoxide synthetase in Benzidine and 5-nitrofuran – induced kidney and bladder carcinogenesis. In: *Prostaglandins and Cancer (Eds., Powles et al.)*, p. 123: Alan R. Liss Inc., New York.